# Harmonization of postmortem donations for pediatric brain tumors and molecular characterization of diffuse midline gliomas

**DOI:** 10.1038/s41598-020-67764-2

**Published:** 2020-07-02

**Authors:** Madhuri Kambhampati, Eshini Panditharatna, Sridevi Yadavilli, Karim Saoud, Sulgi Lee, Augustine Eze, M. I. Almira-Suarez, Lauren Hancock, Erin R. Bonner, Jamila Gittens, Mojca Stampar, Krutika Gaonkar, Adam C. Resnick, Cassie Kline, Cheng-Ying Ho, Angela J. Waanders, Maria-Magdalena Georgescu, Naomi E. Rance, Yong Kim, Courtney Johnson, Brian R. Rood, Lindsay B. Kilburn, Eugene I. Hwang, Sabine Mueller, Roger J. Packer, Miriam Bornhorst, Javad Nazarian

**Affiliations:** 1 Center for Genetic Medicine Research, Children’s National Hospital, Washington, DC USA; 2 Brain Tumor Institute, Children’s National Hospital, Washington, DC USA; 3 Department of Pediatric Oncology, Dana-Farber Cancer Institute, Boston, MA USA; 4 Department of Pathology, Children’s National Hospital, Washington, DC USA; 5 Center for Cancer and Immunology Research, Children’s National Hospital, Washington, DC USA; 6 PTC Therapeutics, South Plainfield, NJ USA; 7 Center for Data-Driven Discovery in Biomedicine, Children’s Hospital of Philadelphia, Philadelphia, PA USA; 8 Pediatric Hematology-Oncology and Neurology, UCSF Benioff Children’s Hospital, San Francisco, CA USA; 9 Department of Pathology and Neurology, University of Maryland School of Medicine, Baltimore, MD USA; 10 Feinberg School of Medicine, Northwestern University, Chicago, IL USA; 11 NeuroMarkers PLLC, Houston, TX USA; 12 Department of Pathology, University of Arizona College of Medicine, Tucson, AZ USA; 13 Pediatric Oncology Branch, National Cancer Institute, Bethesda, MD USA; 14 Department of Oncology, Children’s Research Center, University Children’s Hospital Zürich, Zurich, Switzerland; 15 The George Washington University School of Medicine and Health Sciences, Washington, DC USA; 16 Division of Oncology, Children’s Hospital of Philadelphia, Philadelphia, PA 19104 USA

**Keywords:** Neuroscience, Cancer, CNS cancer, Paediatric cancer

## Abstract

Children diagnosed with brain tumors have the lowest overall survival of all pediatric cancers. Recent molecular studies have resulted in the discovery of recurrent driver mutations in many pediatric brain tumors. However, despite these molecular advances, the clinical outcomes of high grade tumors, including H3K27M diffuse midline glioma (H3K27M DMG), remain poor. To address the paucity of tissue for biological studies, we have established a comprehensive protocol for the coordination and processing of donated specimens at postmortem. Since 2010, 60 postmortem pediatric brain tumor donations from 26 institutions were coordinated and collected. Patient derived xenograft models and cell cultures were successfully created (76% and 44% of attempts respectively), irrespective of postmortem processing time. Histological analysis of mid-sagittal whole brain sections revealed evidence of treatment response, immune cell infiltration and the migratory path of infiltrating H3K27M DMG cells into other midline structures and cerebral lobes. Sequencing of primary and disseminated tumors confirmed the presence of oncogenic driver mutations and their obligate partners. Our findings highlight the importance of postmortem tissue donations as an invaluable resource to accelerate research, potentially leading to improved outcomes for children with aggressive brain tumors.

## Introduction

Brain tumors are the most common childhood malignancy, and are the leading cause of cancer-related mortality in children^1,2^. Gliomas are the most common type of pediatric brain tumors^3^. Among these, pediatric high grade gliomas are the most aggressive, with an average 5-year overall survival of only 20%. The high rate of mortality poses a need for improved understanding of tumor biology, so this information can be used to develop new targeted therapies and design effective clinical trials.

Discoveries of epigenetic and molecular subgroups of pediatric high grade glioma are primarily based on genomic studies conducted on tumors collected at the time of primary diagnosis, recurrence, or post-mortem. Hemispheric, non-midline GBMs are typically surgically resected at diagnosis, and exhibit intratumor heterogeneity and plasticity, where glioma stem cells resemble neural progenitor-, oligodendrocyte precursor-, astrocyte and mesenchymal-like states^4^. Tumor driver mutations found in pediatric glioblastoma (GBM) include *BRAFV600E* point mutations, alterations in *NF1, TP53, EGFR*, and *PDGFRA* with or without concurrent *CDKN2A/B* deletions, and histone 3.3 G34R/V mutations^5–10^. Tyrosine receptor kinase (TRK) fusions (*NTRK1, NTRK2, NTRK3, ALK, ROS, MET*) are also seen in up to 50% of infant GBM^8,11,12^.

In contrast to hemispheric gliomas, diffuse midline gliomas (DMGs) are rapidly growing tumors that arise in midline region of the brain and are typically not amenable to surgical resection. DMGs affect over 1,000 children every year in the United States and Europe with a 5-year overall survival of only 2%^13^. Previously, the lack of surgical specimens, coupled with the rarity of this disease, led to a paucity of tissue specimens available for molecular studies and the generation of preclinical models. However, over the past 8 years, there has been a surge in the molecular understanding of DMG tumors including the identification of novel oncohistones, the putative cell of origin, and mutational landscape^5,8–10,14–20^. Our improved understanding of DMG tumor biology occurred, in large part, due to the increased availability of tumor tissue through rare diagnostic biopsies^21,22^ and more frequently from the donation of postmortem specimens^23^.

Oncohistones are genetic driver events found in over 60% of pediatric high grade gliomas, where histone 3.3 G34R/V mutations primarily occur in hemispheric gliomas and histone 3 K27M (H3K27M) mutations occur in midline (brainstem, thalamus, spinal cord) brain anatomical location^9,10,24^. The predominant presence of heterozygous K27M mutations in genes encoding the histone 3 variants 3.1, 3.2, and 3.3 (*H3F3A, HIST1H3B/C, HIST2H3C)* in midline gliomas led to the revised classification of “H3K27M diffuse midline glioma” by the World Health Organization in 2016^25^. Diffuse intrinsic pontine glioma (DIPG), which originates in the pons of the brainstem, belongs to the H3K27M DMG classification of tumors.

We and others have shown that H3K27M is thought to arise as an initial oncogenic driver event in midline gliomas, followed by secondary genomic alterations in cell cycle regulatory and growth factor signaling pathways^26–28^. The putative cell of origin of H3K27M DMG is an oligodendrocyte precursor-like cell, which is highly proliferative, capable of self-renewing, and exhibits high expression of *PDGFRA*^14^. Among H3K27M DMGs, H3.3 K27M mutated tumors occur in all midline locations, co-occur most frequently with *TP53* pathway alterations, are resistant to radiotherapy, and harbor the worst overall survival (median 11 months) compared to H3.1 K27M, and H3 wild type (WT) tumors. DMG tumors harboring H3.1 K27M are mainly restricted to the brainstem, and occur in younger children (median age of 5 years), and are commonly comprised of *ACVR1* mutations^10,20,29–31^. Recent findings have shown rare H3 WT DMG tumors overexpress *EZHIP*, which is an oncohistone-mimic of H3K27M, and these patients have an overall survival similar to H3.1 K27M tumors (median 15 months)^20,32^.

Unlike diagnostic biopsies, postmortem brain tissue can be used to analyze tumor dissemination within brain parenchyma, in order to identify spatial genomic signatures and clonal evolution across tumor cell migration^13,17,27^. Recently, upfront biopsies matched with postmortem tissue showed longitudinal molecular changes across the tumor genome^33^, allowing for a better understanding of tumor evolution at progression. Analysis of postmortem specimens has also expanded our understanding of DMG tumor heterogeneity, highlighting histologic and molecular heterogeneity within the tumor, and the implications this can have on responses to treatment^34^. Finally, postmortem specimens have been a major source of existing preclinical disease models as outlined previously by us^23^ and others^35–37^.

Existing postmortem tissue donation literature describes the consenting process, socioeconomic/religious factors affecting tissue donation, generation of preclinical models, and sample integrity of postmortem tissue^38,39^. However, a comprehensive protocol outlining the harmonized effort of coordinating a successful postmortem donation, and details of whole brain and spinal cord specimen processing that allows for maximized utility of tumor and healthy brain regions for molecular analyses and generation of preclinical models, is still needed.

The postmortem tissue donation program at Children’s National Hospital (CNH) was established in 2010, with the aim of acquiring DMG tumor specimens to allow for further understanding of disease biology. Over time, the CNH postmortem donation program has evolved to include other brain tumor specimens upon family’s request. In this study, we first describe our experience in coordinating postmortem donations for pediatric central nervous system (CNS) cancers and outline a comprehensive protocol that provides detailed information on logistics, organization and execution of the donation process. We further outline examples of how donated specimens were utilized for downstream biological studies of the disease. Our comprehensive protocol allows for the compilation and annotation of relevant clinical information, the collection and processing of whole brain, spinal cord, biofluids (blood and cerebrospinal fluid (CSF)), and germline controls for molecular characterization, and the generation of preclinical models using post-mortem specimens.

## Results

### Coordination and processing of postmortem donations

**Figure 1:**
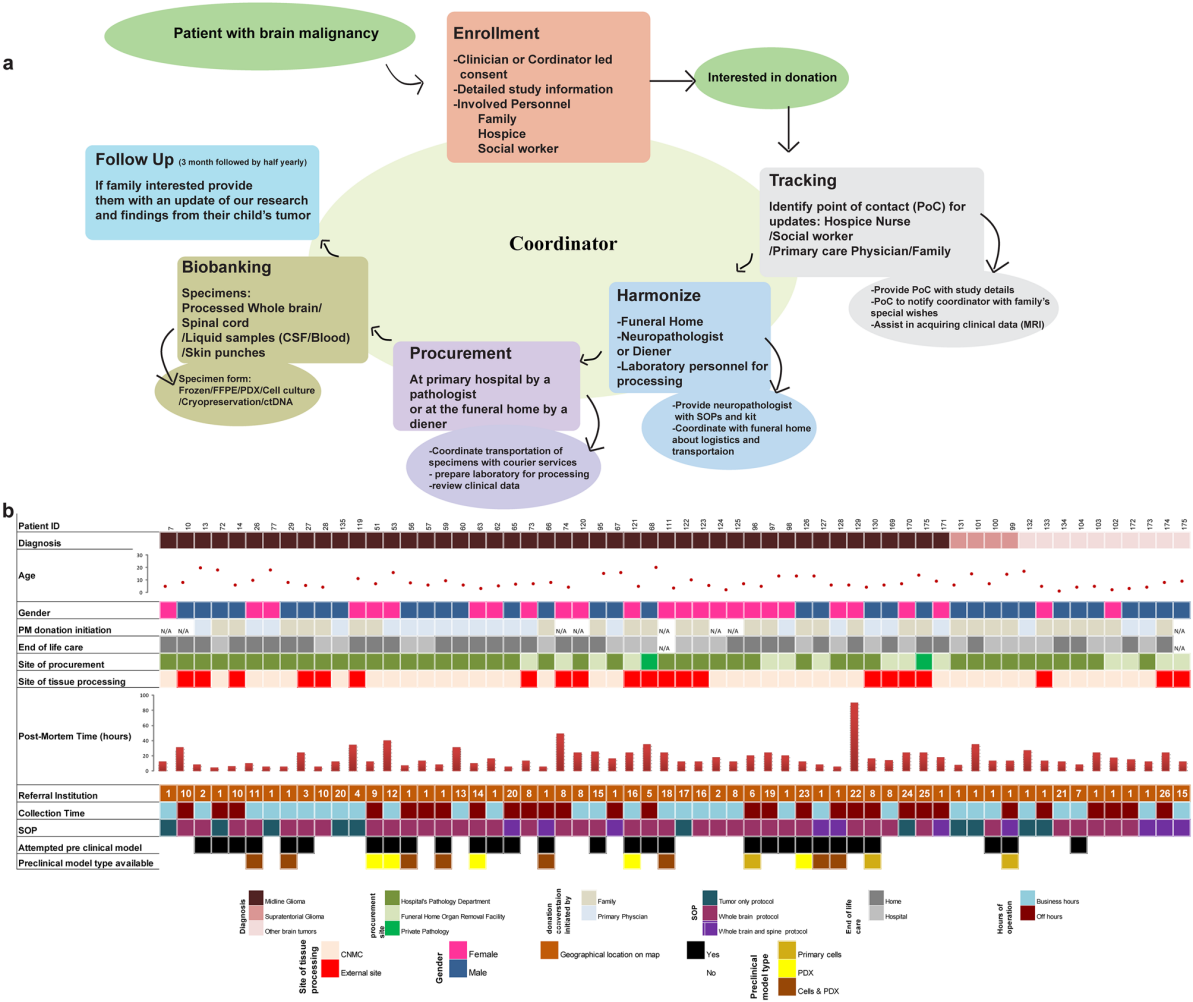
Coordination characteristics of postmortem donation program at CNH. (**a**) Flow chart of personnel and steps involved for coordination. (**b**) Patient demographics, types of brain tumors collected, logistics, and generation of postmortem preclinical models. Flow chart graphics were made using Adobe Illustrator.

Since its establishment in 2010, the CNH tissue donation protocol has evolved to include the following cascade of events, which allow for maximum efficiency of sample procurement and processing while minimizing burden on the patient’s family in the immediate postmortem period: (1) identification of a patient/family interested in donation in partnership with other institutions, (2) consent process, (3) patient enrollment, (4) assignment of a point of contact who can provide updates on the patient’s status to the clinical coordinator, (5) collection of patient’s clinical data such as drug treatment, surgical procedures and MR images, (6) coordination with the funeral home, (7) identification of a neuropathologist or diener for specimen acquisition, (8) coordination with laboratory personnel responsible for the collection and biobanking of tissue and biofluid specimens, and (9) follow up with the family (Fig. 1a). The CNH enrollment form (CNH postmortem tissue donation program) and steps from the CNH standard operating procedure (SOP) are outlined in Supplementary Note 1.

We have coordinated over 70 cases and collected postmortem donations from 60 pediatric CNS cases including diffuse midline glioma (n = 46), supratentorial glioma (n = 4), and other brain cancers (n = 10) (Fig. 1b). Information about whether the patient’s family or the treating physician/care team initiated discussion about postmortem donation was collected for a majority (n = 52) of cases (Fig. 1b). In these cases, discussion was initiated by the patient’s family 58% of the time (n = 30). Patient ages ranged from 0.9 to 20 years, with 57% (n = 34) male and 43% (n = 26) female subjects. More than half of patient deaths occurred at home (n = 34; 57%). The majority (n = 39; 65%) of cases were processed at CNH (Fig. 1b) while the remaining cases were processed at either referral institutions or private pathological facilities. The time from postmortem donation to tissue processing ranged from 4 to 96 h with a median of 13 h and an inter quartile range (IQR) of 14 h (Fig. 1b). Among the 60 cases, 40% (n = 24) were collected from subjects that received primary care at CNH and the remaining were referred from external institutions. Postmortem donations were coordinated for subjects from 25 external institutions across the United States, Canada and Europe (Supplementary Fig. S1).

**Figure 2:**
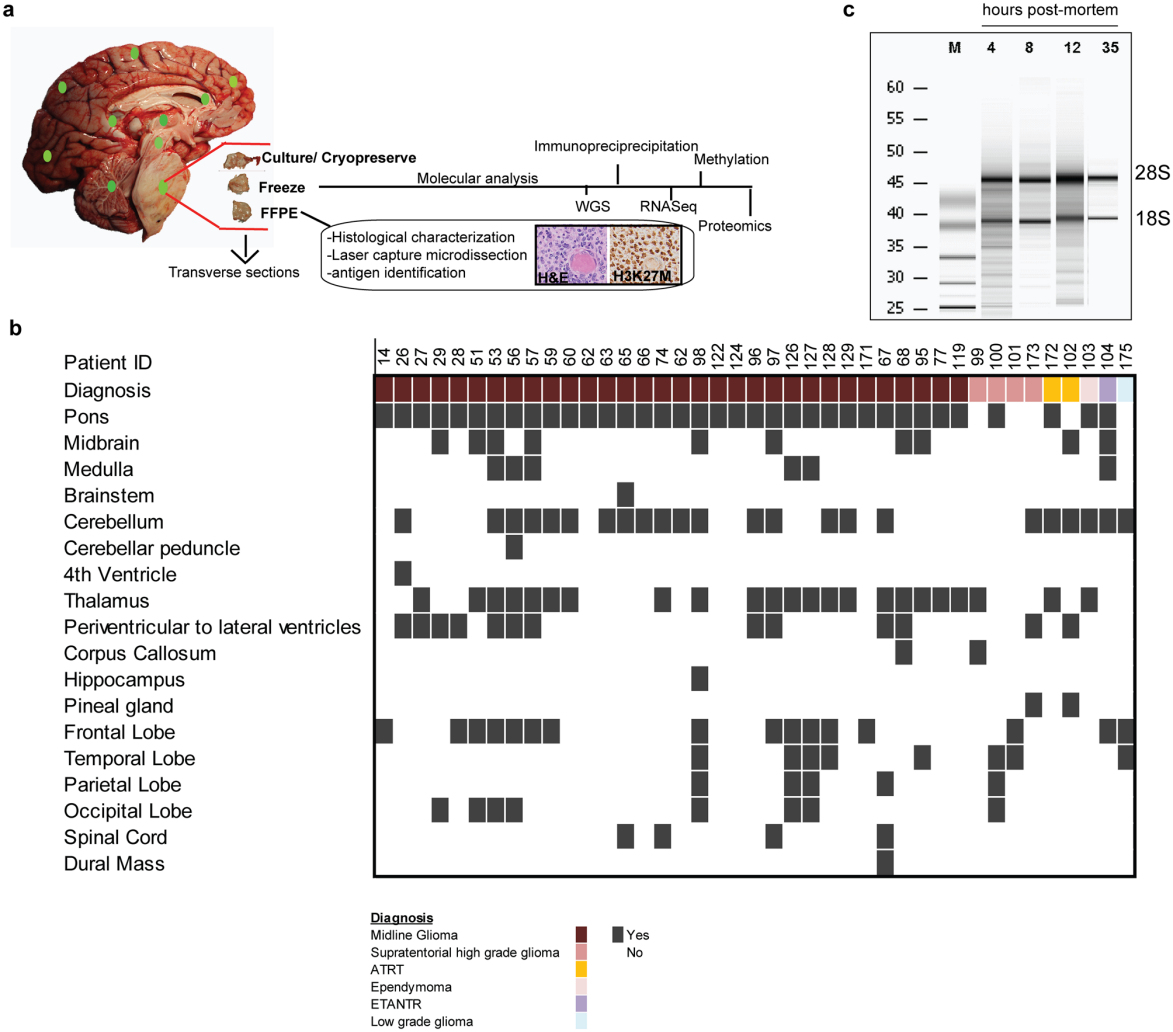
Specimens collected, comprehensive whole brain processing, and utilization of specimens for preclinical models. (**a**) Detailed figure of processing in a patient with DMG using different platforms and utilization of tumor and healthy regions for frozen, FFPE, preclinical model generation (cell culture or xenograft) and DMSO preservation of whole brain. (**b**) RNA integrity was not affected by postmortem tissue collection time in DMG tumors. (**c**) Tissue collected for DMSO preservation representing multiple neuroanatomical locations for future generation of preclinical models. Graphics were made using Adobe Illustrator.

The initial CNH comprehensive postmortem protocol focused on collection of whole brain, blood and CSF, with an emphasis on cryopreservation of the primary tumor. Over time this has been amended to include the collection of additional specimens including spinal cord tissue and a skin punch, which serves as a germline control and cryopreservation of multiple brain regions (Fig. 2, Supplementary Fig. S2a and S2b). Details of processing and standard operating procedures for the current comprehensive postmortem protocol are summarized in Supplementary Note 2. A comprehensive whole brain protocol was conducted in 65% (n = 39) of subjects, while a comprehensive whole brain and spine processing protocol was performed for 17% (n = 10) of subjects. For a few cases (n = 10) tissue procurement and processing were conducted using a modified version of our protocol due to resource limitations (Fig. 1b; Supplementary Note 3). In one instance we collected tumor only based on the family’s request.

### Postmortem specimen integrity and utility for genomic profiling studies

Tissue from primary tumor was frozen for all cases to allow for genomic profiling studies (Fig. 2a). RNA integrity, as shown by intact ribosomal subunit bands, did not appear to be affected by longer tissue processing time (e.g. 35 h) (Fig. 2c). DNA and RNA quality were sufficient to successfully conduct whole genome/exome sequencing (n = 34), methylation (n = 48), and RNA profiling studies via RNAseq and gene expression arrays (n = 8) on tumors collected at postmortem (Supplementary Fig. S2c). The remaining samples have been submitted, with results pending.

### Preclinical models derived from postmortem tissue recapitulate disease

**Figure 3:**
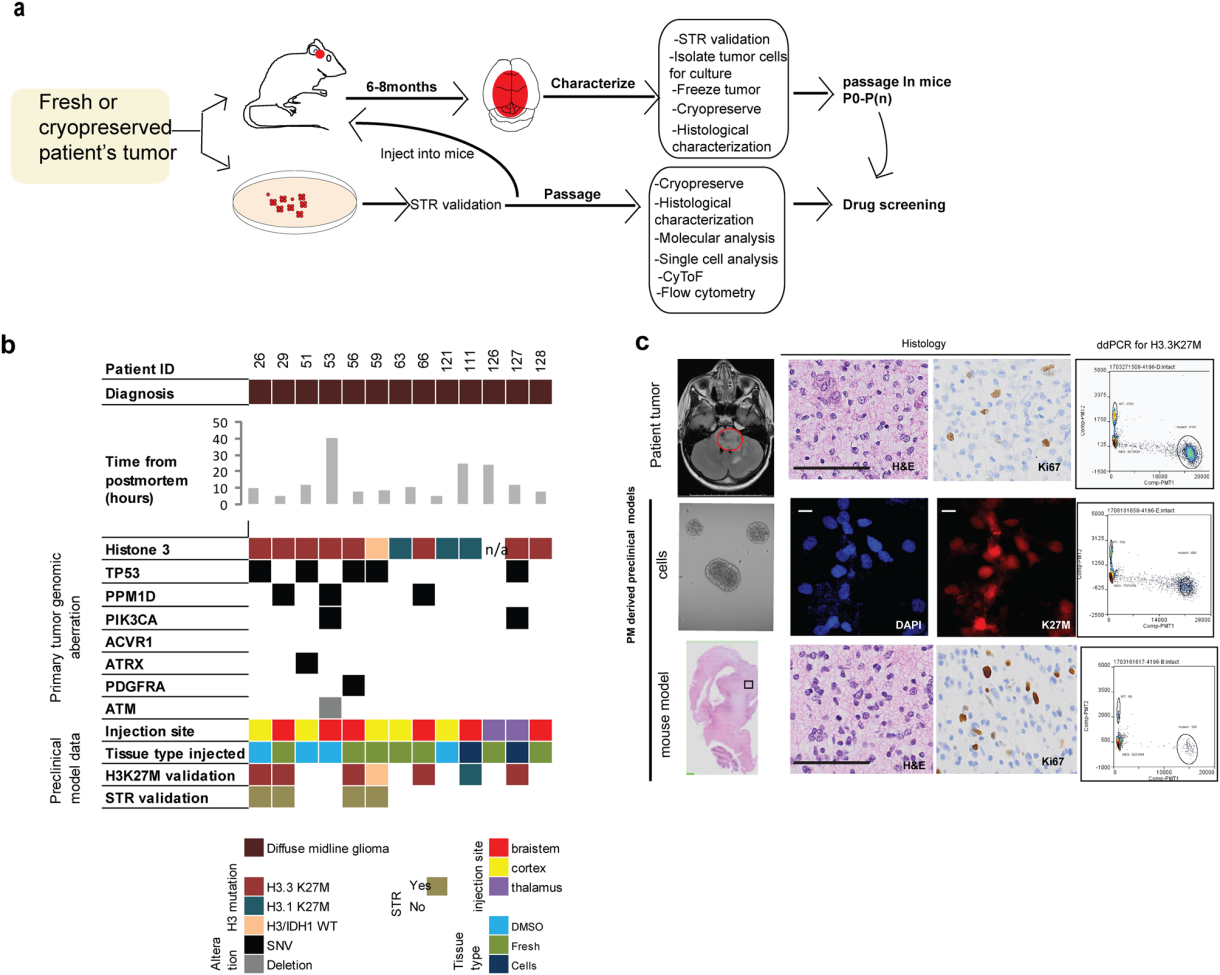
Characterization and fidelity of DMG preclinical models derived from postmortem tissue. (**a**) Schematic for generation of preclinical models (xenografts or primary cells) from fresh or DMSO cryopreserved tissue. (**b**) Oncoplot showing preclinical models generated and genomic aberrations of primary tumor obtained from WGS. (**c**) Characteristics of primary tumor and a matched primary cell line and xenograft model derived from postmortem tissue harboring H3.3K27M. *Scale Bar* = 100 µm. Schematic graphics were created using Adobe Illustrator.

Freshly obtained tumor specimens were processed for cell culture and orthotopic intracranial injection to allow for the direct generation of preclinical models when possible (Figs. 1b, 3a,b). In addition, tissue from primary tumor (n = 41), multiple brain (n = 34) and spinal cord (n = 3) regions were DMSO cryopreserved during post mortem processing (Fig. 2b) to allow for future development of preclinical models.

Forty-one preclinical (PDX and primary cell cultures) models were attempted and twenty-four models were generated (58%) utilizing postmortem tumor samples (Fig. 1b). Generation of a primary neurosphere cell line was defined as successful if the primary cells were passaged a minimum of five times (Fig. 1b). Primary neurosphere cell lines were successfully generated 44% (11/25) of the times this was attempted. Successful primary neurosphere cultures were either derived from fresh tumor tissue collected at postmortem (n = 7) or from PDX tumors (n = 4). Most of the primary neurosphere cell lines were derived from DMG patients (n = 10) and retained their histone 3 mutation or wildtype status as assessed by immunofluorescence and droplet digital PCR (ddPCR) (Fig. 3c). The other primary neurosphere model was derived from supratentorial glioma tissue harboring BRAF V600E mutation (Fig. 1b, patient 99).

PDX model generation was performed using either brainstem, thalamus, or cortex as the primary site of injection (Fig. 3b). The site of injection was decided based on the type of tumor as well as availability of mice at the time of injections. PDX models were either generated from fresh postmortem tissue, cryopreserved tissue, or primary neurosphere cells (Figs. 2b, 3b). PDX models of DMG were defined as successful if they were passaged at least once, and represented primary tumor characteristics by hematoxylin and eosin (H&E), Ki67, and H3K27M (if applicable) analysis (Fig. 3c). PDX models were successful 76% (n = 10/17) of the times this was attempted. Cryopreserved tissue was successfully used to generate PDX models 50% (4/8) of the time this was attempted, while fresh tissue was successful 75% (9/12) of the time, and neurosphere cell lines were successful 100% (2/2) of the time. Most PDX models were generated from samples that harbored DMG mutation profiles containing combinations of H3.3K27M/PPM1D/PIK3CA, H3.1/ACVR1/TP53, and H3WT (Fig. 3b). The fidelity of these PDX preclinical models was validated by comparing the short tandem repeat (STR) and H3K27M status to the parent tumor specimens (Fig. 3b,c). Postmortem collection time did not seem to influence successful generation of a PDX model, as PDX models were generated with samples collected at a range of 5–40 h (median of 10 h) (Fig. 1b).

### Patterns of tumor migration and molecular characterization of H3K27M DMG

**Figure 4:**
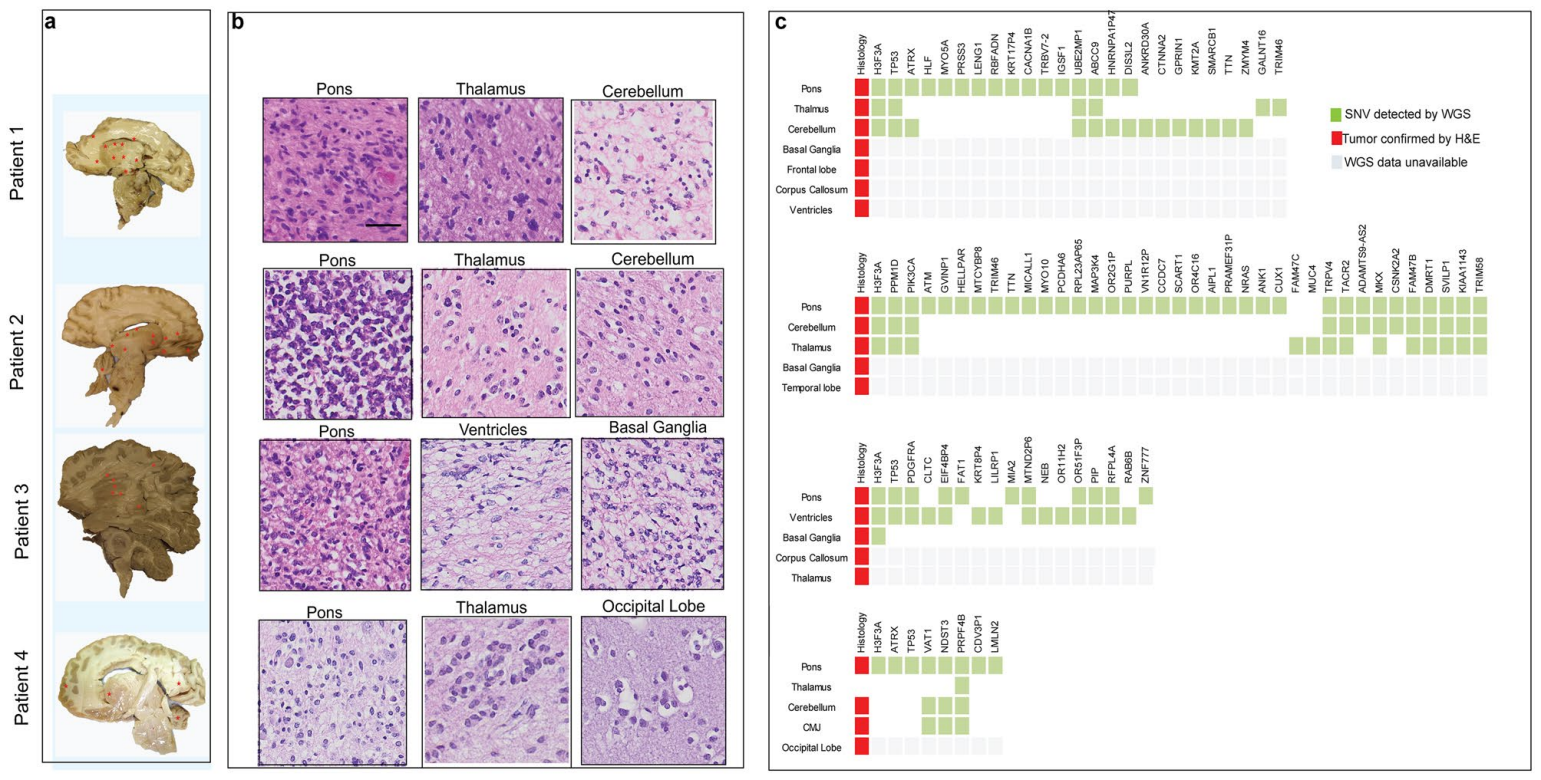
Identification of patterns of tumor dissemination in H3K27M DMG. (**a**) Mid-sagittal histological characterization for identification of tumor dissemination (shown by asterisk*) as assessed by a neuropathologist. (**b**) H&E images of specific locations confirming presence of tumor. (**c**) Mutational profile identified in primary tumor and other neuroanatomical locations show affected oncogenic pathways (not all locations mentioned in the oncoplot have tumors as per histology or mutational profile). *Scale Bar* = 100 µm Final figure was combined on Adobe Illustrator.

Neuropathological review of four midsagittal sections of whole brain H3K27M DMG specimens revealed tumor dissemination from pons to other infra- and supratentorial regions in all cases (Fig. 4a). Consistent with previous studies^26,28,40^, three of the four H3K27M DMG patients (patients 1–3) had disseminated tumor cells located in other midline locations such as the thalamus, basal ganglia, the periventricular regions, and cerebellum (Fig. 4a,b). In patients 1, 2 and 4, tumor cells also infiltrated distant locations such as the frontal, temporal, and occipital lobes, respectively.

To further understand the mutational profile of disseminated tumors, whole genome sequencing (WGS) on multiple regions of tumor and healthy brain of these four patients diagnosed with H3K27M DMG was performed. WGS was also performed on blood when available to serve as germline controls. The primary mutation, *H3F3A* p.K27M was detected in all four patients’ primary tumors and in three patients’ disseminated tumor sites (6 of 19 evaluated sites) (Fig. 4c). All four patients also harbored the classical association of H3K27M with mutations in genes encoding *TP53* or *PPM1D,* affecting the TP53 cell cycle regulatory pathway. Other recurrent mutations in primary and disseminated H3K27M DMG tumors included *ATRX, PIK3CA,* and *PDGFRA* (Fig. 4c). Interestingly, patient 2 harbored a mutation in *ATM* only in the primary pontine tumor, but not in disseminated tumor sites.

Remarkably, disseminated tumors in the cerebellum, cervico medullary junction (CMJ) and occipital lobe of patient 4 did not harbor the H3K27M or other partner mutations found in the primary pontine tumor (Fig. 4c). However, the disseminated tumors harbored two other subclonal mutations (*VAT1, NDST3*), and one clonal mutation (*PRPF4B*) in common with the pontine tumor. Similarly, disseminated tumors in patients 1, 2, and 3 harbored subclonal mutations that were not detected in the pons. Strikingly, the cerebellar disseminated tumor in patient 1 contained seven subclonal mutations that were absent in the pons. Among these were mutations in genes encoding a cell adhesion gene (*CTNNA2),* and the chromatin regulators *KMT2A* and *SMARCB1*, known to be altered in other types of pediatric cancers^41–43^.

### Patterns of treatment and immune response in H3K27M DMG postmortem samples

Postmortem tissue was also analyzed to determine the effects of treatment on tumors and healthy brain regions. Patient 53 received radiation and convection enhanced delivery (CED) of 8H9. In order to assess the effects of this specialized treatment on the pontine tumor and surrounding tissue, a mid-sagittal slice of the whole brain was obtained during post-mortem processing (Supplementary Fig. S3). The mid-sagittal slice was fixed as a whole, and formalin fixed paraffin embedded (FFPE) blocks were generated and stained for hematoxylin and eosin (H&E). Upon neuropathological review, pontine locations that had received CED treatment had decreased cellularity compared to other locations within the brainstem (Supplementary Fig. S3a). Further examination of mid-sagittal section revealed tumor dissemination to other untreated regions of the brain such as thalamus and occipital lobe.

Similarly, whole brain postmortem specimens were used to identify immune cells within the primary tumor (n = 3) and the primary and extended tumor (n = 3) from DMG cases. CD45 (leukocyte common antigen) immunohistochemistry (IHC) staining revealed infiltrating immune cells intermixed with tumor cells (which were identified through H&E and H3K27M IHC staining) in both the primary and extended tumors (Supplementary Figs. S3b,c).

## Discussion

Despite numerous basic, translational, and clinical research efforts over the past few decades, a more comprehensive understanding of brain tumor biology is still needed to develop novel effective therapies. Postmortem tissue donations serve as an invaluable source of end-stage disease biology, which plays a pivotal role in unravelling the dynamic and complex evolution of these aggressive tumors. In this paper, we outline the CNH comprehensive postmortem tissue processing protocol and describe how this protocol can be used to improve our understanding of the molecular, histologic, and immunologic features of rare tumor samples.

In order to maximize the scientific knowledge gained from postmortem tissue samples, there are a multitude of steps that need to be followed (Fig. 1a). The most crucial step in this process is the identification of families that are interested in postmortem donation. Of the 60 cases included in this analysis, approximately half of the discussions regarding postmortem donation were initiated by the patient’s family. This is in part due to programs such as the Swifty Foundation’s ‘Gift From A Child’, which was developed to provide education regarding the donation process to researchers and families, making it easier for prospective families to donate. Programs like these that promote understanding of the significance of donations and improve awareness of the importance of this initiative, ultimately can lead to an increase in families’ interest and discussion about postmortem donations.

After interested patients and families are identified, they are consented and enrolled onto a postmortem tissue study protocol. Consenting to postmortem donation is dependent upon multiple factors: a family’s interest in furthering research efforts, a family’s desire for clarity regarding complications associated with the child’s diagnostic and treatment course, family’s stage of grief, and cultural/religious affiliations. All these factors must be considered when engaging a family, to ensure that their wishes are being acknowledged throughout the entire process. Once a family has consented, the research coordinator plays a major role in synchronizing and updating all personnel and teams involved in the donation process including the family’s point of contact (e.g. family member, hospice nurse, or social worker), neuropathologist, funeral home, and the laboratory team in charge of processing tissue.

Despite careful planning, there are occasional challenges in the coordination and execution of postmortem donation. In some cases, particularly during off-hours and holidays, the pathologist and/or laboratory personnel are not available for immediate tissue processing, limiting the center’s ability to accept a post-mortem donation. A diener is a trained autopsy technician who assists a pathologist with dissection during an autopsy. To address this challenge, dieners can be hired for tissue procurement when a neuropathologist is not available. In order to execute the comprehensive whole brain and spine processing protocol, it typically requires four to six lab personnel for efficient freezing and fixing of tissue, as well as processing of fresh tissue for DMSO and the generation of preclinical models. During times when there are insufficient staff members available for processing, a modified SOP protocol, that allows for rapid processing of samples with fewer personnel, can be used instead (Supplementary note 3).

Preservation of tissue from multiple regions across the whole brain and spinal cord allows for the generation of preclinical models that represent the primary tumor and disseminated tumors. MR images are important for locating primary and disseminated tumors during tissue processing. Whole brain processing of a multi-focal diffuse low grade glioma (patient 175) proved to be challenging as tumor was not visible by gross examination of postmortem tissue. While prior MRI reports were obtained, the most recent MR images were not available at the time of postmortem, precluding maximally efficient tumor sampling. In contrast, availability of MR images (patient 65) helped to determine tumor dissemination to the cerebellum and spinal cord. Therefore, the donation process was modified to allow for collection of both primary and metastatic sites which were preserved in DMSO to be used for future pre-clinical models. Subsequently, gross and histological examination of the samples by a neuropathologist confirmed tumor infiltration of the cerebellum, hippocampus and spinal cord. Therefore, it is critical to use an SOP that includes the use of the latest MR images in order to prioritize tumor locations for collection.

The accuracy of genetically and histologically faithful preclinical models is important for translation of novel agents into the clinic. Preclinical models were derived from tumors representing various molecular subtypes of DMG (H3.3K27M/PPM1D/PIK3CA, H3.1/ACVR1/TP53, H3WT) and the fidelity of these preclinical models was shown by mutation analysis and histological characterization. Importantly, postmortem collection time did not seem to influence successful generation of a PDX model, as PDX models were generated with samples collected at a range of 5–40 h. In our experience, xenograft model generation was more successful than primary cell cultures. This could be due to tumor cell microenvironment requirements that are naturally present in a mouse. Our study also shows that utilizing fresh tissue is more favorable than cryopreserved tissue for the generation of PDX models in concordance with previous studies^44^. However, this observation needs to be validated in a larger cohort of samples.

Postmortem tumor tissue is also useful for the histologic and molecular analysis of regions of tumor dissemination within the brain parenchyma. In our analysis of four patients with disseminated H3K27M DMGs, WGS sequencing revealed clonal and subclonal mutations within the primary and disseminated tumors. One patient harbored a mutation in *ATM* only in the primary pontine tumor. For this patient, the key genomic aberration in the primary pontine tumor, which was absent in the disseminated tumors, may either be important for tumor initiation and maintenance but not tumor migration, or was a passenger mutation that was no longer present in the disseminated tumor. Three patients also harbored subclonal mutations that were absent from the primary pontine tumor, but present in disseminated tumors. The acquisition of these subclonal mutations may have been driven by specific microenvironment changes that promote adaptations to aid in the process of tumor migration. Future mechanistic studies are required to understand the role of clonal and subclonal mutations found within specific tumor sites (primary and disseminated).

Improved knowledge of the long- and short-term effects of treatment is necessary to determine why therapy is not successful. Molecular and histologic analyses of postmortem tissue can provide essential information about the combined effects of radiation, chemotherapy, and other molecularly targeted or immune based therapies on both tumor and healthy brain cells. This information can especially be helpful for patients with DMGs who undergo a variety of therapies on clinical trials. In this study, we used sagittal whole brain sections to analyze the effect of CED therapy on a H3K27M DMG tumor. This analysis revealed decreased cellularity in the region where CED therapy was delivered, indicating that the treatment was successful in this area. However, other areas of the brain that had tumor disseminated did not seem to be affected by therapy. Additional studies using a similar approach can help inform clinical trials for CED or other therapeutic agents, to optimize treatment delivery and effect. We also showed that whole brain postmortem samples can also be used to identify immune cells within the tumor and disseminated tumor sites. Additional studies, to understand the immune response following different treatment modalities, with a focus on immunotherapy, are still needed.

Specimens collected through our program have been shared with the research community leading to collaborative, impactful biology studies^17,35,45–47^. Furthermore, upon consent, all whole genome- and RNA-sequencing data generated from our postmortem program will continually be deposited and shared through the platform OPEN DIPG via the Gabriella Miller Kids First Research Act, an international collaboration led by Children’s Brain Tumor Tissue Consortium (CBTTC). This platform allows for a large collection of sequencing data that can be mined by the scientific community to improve the understanding of disease biology and identify novel targets of therapy. In addition to sharing information with the medical community, it is important to engage with families in discussion about the impact of their donation, particularly when families express interest in receiving this information at the time of the donation. In the CNH postmortem program, unless they request not to receive updates, families are provided with information regarding publications or other mechanisms by which their donation contributed to the scientific field during specific time intervals (Fig. 1a). Increased awareness of postmortem donations coupled with successful processing and maximized utility of postmortem specimens are critical for accelerating research and improving the outcome of rare pediatric brain tumors.

## Methods

### Patient consent and clinical information

All experimental protocols were approved by Children’s National institutional review board (IRB), protocol #Pro00001339 (NCT01106794). Consent to the postmortem tissue donation program was obtained by the patient’s primary physician as per guidelines and regulations provided by our institutional IRB. The topic was discussed with the patient’s family either when patient’s health declined or earlier if the family broached the subject. Post-mortem donation consenting was primarily conducted during hospital visits; although consent could also be obtained via phone call should a patient’s sudden decline or distance inhibit face-to-face discussion. Informed consent/assent was obtained from the patient and family prior to the patient’s passing, and a guardian later authorized autopsy on behalf of the deceased. During the consenting process, particular emphasis was placed on the issues of confidentiality, samples to be collected, and access to medical records. After a careful reading of the consent, families were asked to voice any questions or concerns regarding the autopsy and potential risks. They then signed the consent and a copy was provided to the family.

The protocol was restricted to children age 2 month—25 years with a cancer diagnosis. Patients consented to a limited autopsy allowing for collection of specimens including whole brain, spinal cord, blood, CSF, and a 5 mm skin biopsy punch. Families were assured that any incisions made would not be visible in the case of an open-casket funeral and that only the samples named in the consent would be collected. Families were given the opportunity to limit the extent of autopsy, retention of samples, long-term access to clinical data, and sharing of clinical data to outside institutions. Medical records review was conducted at the time of the postmortem to document diagnosis, treatments, outcomes, and time points of progression.

### Development of preclinical models

All experiments were carried out following guidelines given by Children’s National Institutional Animal Care and Use Committee (IACUC), approved protocol # 30425. Tissue obtained at postmortem was utilized to generate pre-clinical models as described previously^23^. Briefly, single cell suspension was prepared by digestion of fresh or cryopreserved tissue with DNase I and Collagenase followed by RBC lysis. The cell pellet was then filtered through 70 micron cell strainer to remove non-dissociated tissue and debris. Live cells were counted by trypan blue staining and plated for cell culture or orthotropic injection into NOD-SCID mice. NOD SCID gamma mice used for orthotopic injections ranged from 2 days-3 weeks old. Briefly, mice were anesthetized by hypothermia and the following coordinates were used for the injections into specific locations in the brain (Thalamus- X = 0.3 mm, Y = 3.5 mm, Z = 1.5 mM from bregma, Brainstem- X = 0.8 mm, Y = 5 mm, Z = 1.5 mM from lambda, Cortex-). Injected mice were monitored daily for a week for signs of distress and all procedures were conducted in accordance with CNHS IACUC protocol #30435. Mice were consequently monitored for 12 months post tumor cell injections for symptoms of brain tumors such as lethargy, enlarged head, and abnormal movements. Mice indicating signs of brain tumor development were euthanized, and whole brains were collected and analyzed by immunohistochemistry and molecular analyses for tumor confirmation.

### Immunohistochemistry and immunofluorescence

Formalin fixed paraffin embedded tissue samples were used for immunohistochemistry as previously described^45^. Briefly, slides were deparafinnized at 60 °C for 1 h followed by xylene and ethanol solutions. Slides were then blocked and incubated with primary antibody for 1 h followed by HRP labeled secondary antibody. Cell grown on coated chamber slides were washed in PBC followed by fixation in 10%formalin for 5 min, and permeabilized in 0.1% Triton X-100 for 10 min. Blocking was performed in 1%BSA for 1 h, followed by primary and fluorescent tagged secondary antibodies incubation. Cells were stained with 4′,6-Diamidino-2-phenylindole (DAPI) and imaged.

### Nucleic acid isolation

DNA/RNA was isolated from frozen tissue using Qiagen nucleic acid isolation kits as per manufacturer’s instructions. All DNA samples were quantified using Qubit fluorometer, and RNA integrity was assessed using bioanalyzer, as per manufacturer’s instructions.

### Droplet digital PCR

Genomic DNA was isolated as described above, and subjected to dropletization and PCR amplification, followed by detection of fluorescence signal as described previously^48^. Droplet digital PCR (ddPCR, RainDance Technologies) was performed for *H3F3A* p.K27M mutant and wildtype copies in DNA isolated from cells and PDX models derived from H3K27M postmortem tumors.

### Whole genome sequencing

Whole genome sequencing was performed at Children’s Brain Tumor Tissue Consortium (CBTTC) in collaboration with Nant Health. All samples were processed as described previously^33^. DNA was isolated as described above and was sequenced using HiSeq X 150 bp read length. Samples were sequenced with 90X coverage and somatic variant calling was performed using workflow that has been setup in common workflow language on CAVATICA. The pipeline uses BWA-MEM, SAMBLASTER and finally Strelka was applied for identification of point mutations. Somatic mutations were compared to the Genome Aggregation Database (gnomAD) to control for population genetics, Catalogue of Somatic Mutations in Cancer (COSMIC) for somatic mutations reported in other cancers to call an alteration in our samples as mutation. All calls were made with an mutation allelic frequency of 25% or more.

## Supplementary information

Supplementary file1 (DOCX 30 kb)

Supplementary Figure S1

Supplementary Figure S2

Supplementary file2 (DOCX 23 kb)

Supplementary file3 (DOCX 22 kb)

Supplementary Figure S3

## Data Availability

The dataset(s) supporting the conclusions of this article is(are) available in the CAVATICA repository. The dataset(s) supporting the conclusions of this article is(are) included within the article (and its additional file(s)).
