# Supplementary material for: Harmonization of postmortem donations for pediatric brain tumors and molecular characterization of diffuse midline gliomas
Source: Sci Rep. 2020 Jul 2;10:10954. doi: 10.1038/s41598-020-67764-2 (PMC7331588; doi:10.1038/s41598-020-67764-2)
Supplement: Supplementary file 1 — Supplementary file1 (DOCX 30 kb) [file 41598_2020_67764_MOESM1_ESM.docx]

**Harmonization of postmortem donations for pediatric brain tumors**

**and molecular characterization of diffuse midline gliomas**

Madhuri Kambhampati^1,2*^, Eshini Panditharatna^1,2,3*^, Sridevi Yadavilli^1,2^, Karim Saoud^1,2,^, Sulgi Lee^1,2,15^, Augustine Eze^1,2^, M.I Almira-Suarez^4,15^, Lauren Hancock^2,5^, Erin R. Bonner^1,2,15^, Jamila Gittens^1,6^, Mojca Stampar^1^, Krutika Gaonkar^7^, Adam C. Resnick ^7^, Cassie Kline^8,16^, Cheng-Ying Ho^9^, Angela J. Waanders^10^, Maria-Magdalena Georgescu^11^, Naomi E. Rance^12^,Yong Kim^13^, Courtney Johnson^2^, Brian R. Rood^2,5^, Lindsay B. Kilburn^2,5^, Eugene I. Hwang^2,5^, Sabine Mueller^8,14^, Roger J. Packer^2^, Miriam Bornhorst^1,2#^, Javad Nazarian^1,14,15#^

**Supplementary Note 1**

**CNH Standard Operating Procedure for Autopsy Coordination**

Principal Investigator: ***Dr.*** ***Javad Nazarian, PhD, Center for Genetic Medicine Research***

1. Tracking details
2. Patient Information

**Name:**

**Age:**

**Sex:**

**DOB:**

**CNH PID:**

1. Autopsy Contact Information

**Contact Name:**

**Relationship to Patient:**

**Phone Number:**

**Email Address**:

**Mailing Address:**

**Pathologist Name:**

**Pathologist Email Address:**

**Pathologist Pager Number:**

**Pathologist Office Number:**

**Pathologist Fax Number:**

**Pathologist Affiliated Hospital:**

**Pathologist Mailing Address:**

**Oncologist Name:**

**Oncologist Email Address:**

**Oncologist** **Pager Number:**

**Oncologist Office Number:**

**Oncologist Affiliated Hospital:**

**Oncologist Mailing Address:**

1. Hospice Information

**Hospice Name:**

**Hospice Contact Person:**

**Hospice Contact Role:**

**Hospice Contact Number:**

**Hospice Address:**

**Social Worker Name (if applicable):**

**Social Worker Email Address (if applicable):**

**Social Worker Phone Number (if applicable):**

1. Funeral Home Information

**Funeral Home Name:**

**Funeral Home Contact Name:**

**Funeral Home Number:**

**Funeral Home Fax Number:**

**Funeral Home Mailing Address:**

1. Additional Notes
2. Coordination Procedures to Follow:
3. **For Hospice/Social Work**

- Contact the hospice nurse or social worker:
  - Introduce yourself—let them know that you are the study coordinator
  - Make them aware that the family has consented to donate samples post mortem
  - Talk to them about the patient’s health status – make sure to ask for regular health updates that are given to clinical staff
  - Ask for basic details about the patient including age, date of diagnosis, any past surgeries (e.g. biopsy) or sample collection
  - Obtain consent forms (1339 PM donation and CNH autopsy authorization forms, if not yet signed by the family)
  - Ask about any special wishes/religious matters that you need to be aware of
  - Let them know that you can contact the family if they wish to speak to you
  - Obtain information on the funeral home as soon as the family decides on one – try to do this as early as possible
  - Brief them on how the case is coordinated, making special note of the following points:
    - Typical autopsy time: 1-2 hours
    - You will talk to the funeral home and make them aware of the case, they will transport the patient to Pathology department if needed
    - You will talk to the pathologist and make them aware of the case
    - Research will pay for transportation and autopsy associated charges
    - Let them know that you will send a follow up email to the nurse on the same day (*They should not share your email with the family without asking you.)*

1. **For Funeral Home**

- Contact the Funeral Home:
  - Give them your contact information
  - Give them the patient and family’s name (father or mother’s first name and last name) *(Only if the family has already spoken to them)*
  - Ask about their hours of operation (making sure to emphasize the lab’s weekend availability)
  - If the procurement will occur at the hospital, ask if they know how to get to this hospital, and provide them with instructions if necessary (hospital address, specific instructions e.g. enter through the loading dock, check in at security,enter the morgue)
    1. Ask them to call you when they arrive at the hospital
    2. Ask them their requirement for delivery and taking back the body (funeral homes occasionally request release forms)
  - Ask them what costs will be associated with the procedure
    1. **A formal estimate is needed**
    2. Once the autopsy is complete, request for the invoice to be emailed *(An invoice, not a statement, is required to request the check ).*
  - Obtain the funeral home’s W9 form (latest version available) to add it to the vendor system

1. **For the Donor Family**

- Contacting the Family:
  - Contact the family and introduce yourself. Offer your condolences. *(Note: Only contact the family if the provider/hospice/social has cleared it prior – not all families will want to speak with you)*
  - Assure the family that you have taken care of such cases before, and will make sure the process is well organized and professionally handled.

1. **For Pathology/Diener**

- Contacting the Pathologist/Diener:
  - Inform the pathologist/diener know of the case
  - Depending on his/her availability, provide the hospice nurse with an ETA
  - Contact the study PI if any issues arise.
  - Print a copy of the autopsy authorization and post mortem tissue collection forms for the pathologists before the autopsy is performed

1. **For Lab Personnel**
   - Coordinator must notify all lab personnel about the case details ( e.g. tumor location, estimated time of tissue arrival ) to confirm their availability
   - Depending on the availability of lab personnel, coordinator assigns tasks (tasks involve: tissue processing for molecular analysis and preclinical modeling, note taking/sample cataloguing)
2. **For Outside CNH Referrals**

- Making All Parties Aware:
  - If the hospice nurse is aware of a pathologist, they should provide you with this information
    1. If the hospice nurse is not aware of a pathologist, contact the hospital Pathology departments where the patient has been treated and inform them that the family has consented to donate samples postmortem
  - The clinical coordinator will call the pathologist and inform them that you are coordinating the case, and ask the following questions:
    1. Whether they can they perform the autopsy on weekends or very late/early hours
    2. The name and contact information of the best person for you to contact *(add this information to the top of the first page)*
    3. An address to send the kit (to receive the whole brain overnight)
       1. If the time is not sufficient, Pathology department should have all the necessary items from the kit to ship you the brain
       2. Inform them that you can replace the shipping box later if needed.
    4. The address that you should direct the funeral home to deliver the body
    5. Whether there is a need to contact Security or any other department
    6. Their estimated procedure time
    7. The location of the hospital/site where the procedure will be conducted
- After-hours use of the CNH Morgue:
  - **MAKE SURE THE FUNERAL HOME HAS TAGGED THE BODY WITH TWO FORMS OF IDENTIFICATION** (e.g.. Name and Date of Birth) when the patient is being dropped off
    1. The patient must be signed in
  - Call Admin Department on duty
  - Obtain approval to use a spot in the Morgue
  - Call Security - Make sure they have received approval for Morgue.
  - Instructions to give to funeral home:
    1. Proceed to Main Atrium
    2. Security will provide instructions on how to get to the back entrance to reach the Morgue.
- Death Certificate:
  - Funeral home will provide death certificate if the death occurred at home
    - If the patient passed while at the hospital, the hospital will provide the death certificate.
  - For release of body from the hospital after autopsy, the death certificate needs to be signed by the patient’s physician.
- Shipment of the fresh specimens
  - Coordinate with courier services to receive the fresh sample on the same day
