# Supplementary figures and images for: Harmonization of postmortem donations for pediatric brain tumors and molecular characterization of diffuse midline gliomas
Source: Sci Rep. 2020 Jul 2;10:10954. doi: 10.1038/s41598-020-67764-2 (PMC7331588; doi:10.1038/s41598-020-67764-2)

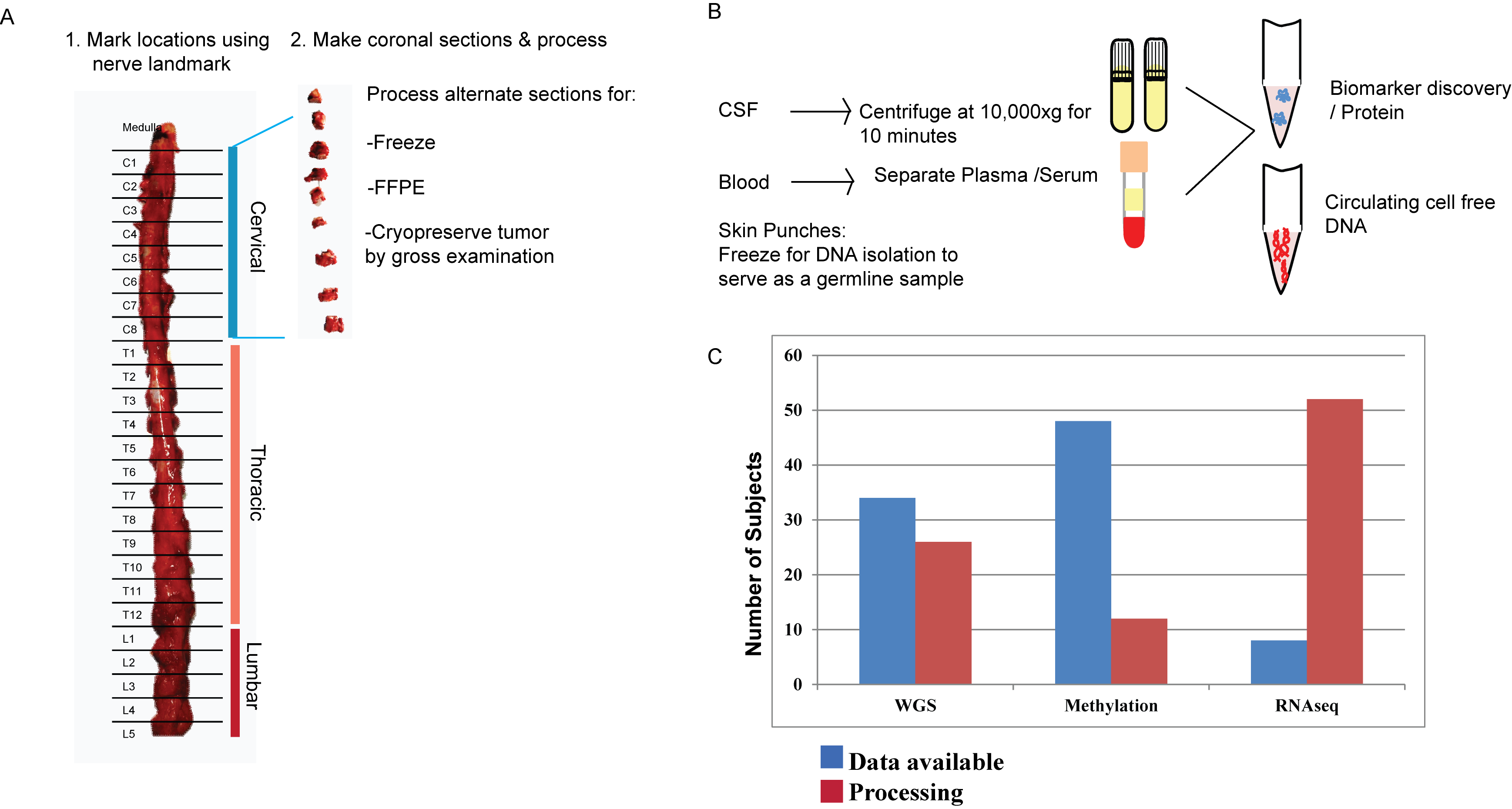

Supplement: Supplementary file 3 — Supplementary Figure S2 [file 41598_2020_67764_MOESM3_ESM.tif]

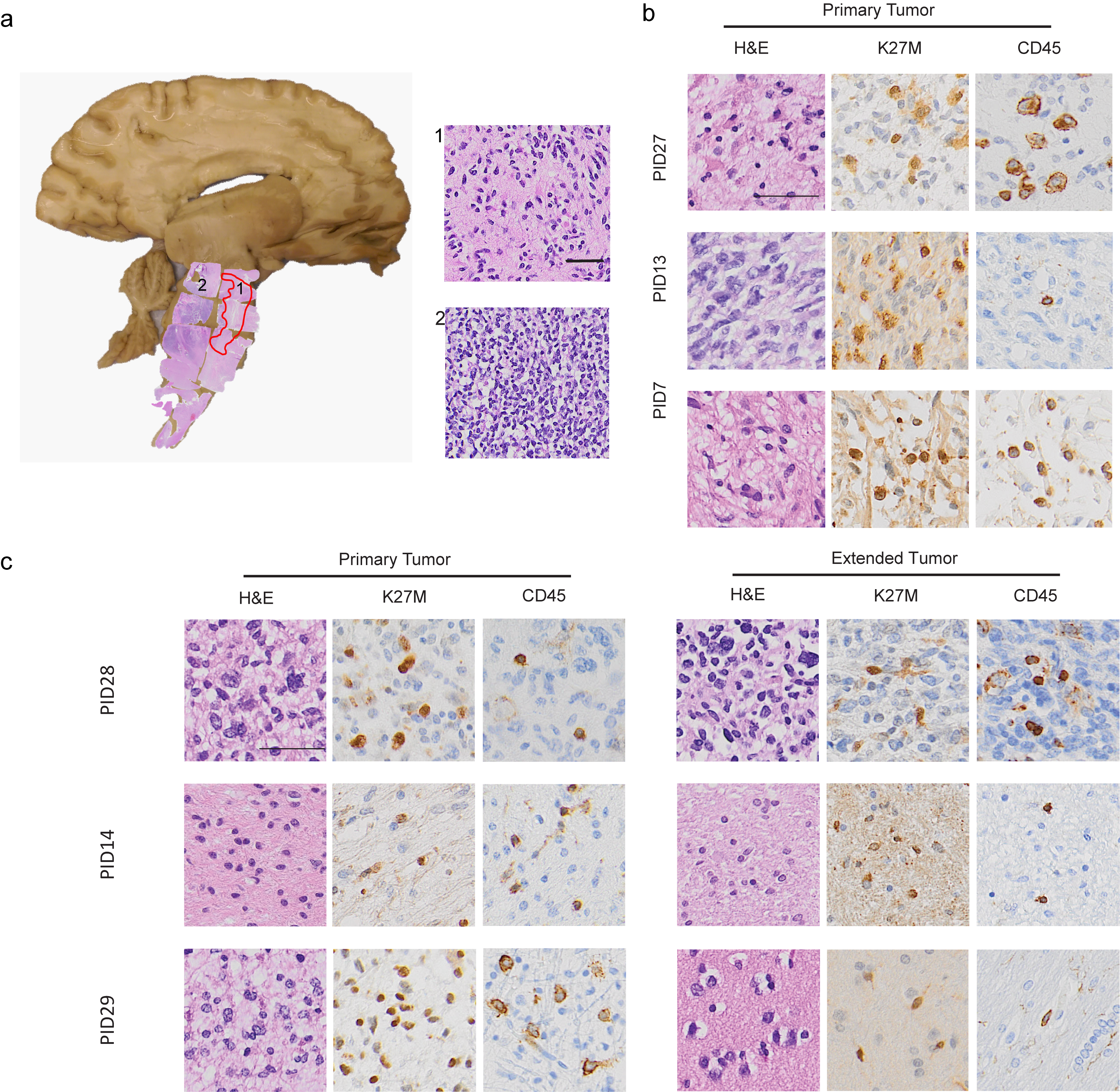

Supplement: Supplementary file 6 — Supplementary Figure S3 [file 41598_2020_67764_MOESM6_ESM.tif]
