# Supplementary material for: Harmonization of postmortem donations for pediatric brain tumors and molecular characterization of diffuse midline gliomas
Source: Sci Rep. 2020 Jul 2;10:10954. doi: 10.1038/s41598-020-67764-2 (PMC7331588; doi:10.1038/s41598-020-67764-2)
Supplement: Supplementary file 4 — Supplementary file2 (DOCX 23 kb) [file 41598_2020_67764_MOESM4_ESM.docx]

**Harmonization of postmortem donations for pediatric brain tumors**

**and molecular characterization of diffuse midline gliomas**

Madhuri Kambhampati^1,2*^, Eshini Panditharatna^1,2,3*^, Sridevi Yadavilli^1,2^, Karim Saoud^1,2,^, Sulgi Lee^1,2,15^, Augustine Eze^1,2^, M.I Almira-Suarez^4,15^, Lauren Hancock^2,5^, Erin R. Bonner^1,2,15^, Jamila Gittens^1,6^, Mojca Stampar^1^, Krutika Gaonkar^7^, Adam C. Resnick ^7^, Cassie Kline^8,16^, Cheng-Ying Ho^9^, Angela J. Waanders^10^, Maria-Magdalena Georgescu^11^, Naomi E. Rance^12^,Yong Kim^13^, Courtney Johnson^2^, Brian R. Rood^2,5^, Lindsay B. Kilburn^2,5^, Eugene I. Hwang^2,5^, Sabine Mueller^8,14^, Roger J. Packer^2^, Miriam Bornhorst^1,2#^, Javad Nazarian^1,14,15#^

**Supplementary Note 2:** SOP for whole brain and spinal cord, SOP for whole brain only, SOP for tumor only, SOP for biofluids and skin punch, SOP for cryopreserving tissue for preclinical modeling or molecular analyses.

1. **CSF Collection**
   1. Expose the brain using the local pathologist’s standards and protocols.
   2. Use a sterile 18- or 21-gauge needle and syringe to collect ventricular CSF. This is best done by gently pulling the hemispheres laterally and aiming the needle laterally from the midline just over the corpus callosum.
      1. Extra CSF can also be collected when available from cisterns during brain removal.
   3. Aliquot CSF and freeze at -80°C.
2. **Brainstem Sectioning**
   1. Remove the brainstem from the cerebral hemispheres at its superior-most aspect (level of substantia nigra).
   2. Make 5mm transverse slices from superior to inferior, leaving the brainstem and cerebellum attached.
      1. Photograph and label the slices from 1 to … (n).
   3. When applicable, collect a piece (3x3x1cm) of tumor from the pons and place in 20mL Hibernate-A media.
      1. Smaller pieces (1cm^3^) of tumor should be collected from other areas of the brainstem and placed in separate vials of Hibernate-A media for cell culture.
   4. Remaining brainstem sections may be frozen or fixed in alternate sections.
      1. Sections allocated for freezing should be placed on aluminum foil and frozen on dry ice or liquid nitrogen prior to storage in a -80°C freezer.
      2. Sections allocated for fixing should be placed in a plastic container and fully submerged in formalin.
3. **Supratentorial Region Sectioning**
   1. Make coronal cuts in the cerebral hemispheres from anterior to posterior.
      1. Photograph and label the slices from 1 to … (n).
   2. If tumor is identified, remove 1cm^3^ and divide the piece into three equal sections for freezing, fixing, and DMSO.
   3. Each supratentorial section will be frozen and fixed alternatively starting from frontal lobe frozen (e.g. section 1-Frozen, section 2-FFPE, section 3-Frozen, and so on)
4. **Spinal Cord**
   1. Identify segments of the spinal cord via holes in the meninges (dura mater particularly) where peripheral nerves connect to the cord.
   2. Section the cord transversally**.**
      1. Alternate freezing and fixing sections of the cord.
5. **Skin Punch**
   1. Preserve 4-5 skin punches (3mm each) collected from the back of the foot, arm or at the incision site.
   2. Freeze at -80°C.
6. **SOP for cryopreserving post mortem tissue**
   1. Collect postmortem tumor tissue measuring ~ 1 cm X 1 cm into Hibernate-A media and immediately transport on ice to the BSL2 cabinet.
   2. Spin the sample at 300x g for 5 minutes and discarded the media.
   3. Transfer the tissue to a sterile 100 mm^2^ petri dish and mince into ~1mm^2^ pieces with a number 10 scalpel.
   4. Add twenty milliliters of HBSS to the petri dish and transfer the tissue to a 50 ml conical tube.
   5. After centrifuging at 300x g for 5 min, remove the supernatant and re-suspend the tissue in TSM complete containing 20% DMSO, aliquot this into 2 ml cryovials and store in a -80 degree freezer.
