# Supplementary material for: Harmonization of postmortem donations for pediatric brain tumors and molecular characterization of diffuse midline gliomas
Source: Sci Rep. 2020 Jul 2;10:10954. doi: 10.1038/s41598-020-67764-2 (PMC7331588; doi:10.1038/s41598-020-67764-2)
Supplement: Supplementary file 5 — Supplementary file3 (DOCX 22 kb) [file 41598_2020_67764_MOESM5_ESM.docx]

**Harmonization of postmortem donations for pediatric brain tumors**

**and molecular characterization of diffuse midline gliomas**

Madhuri Kambhampati^1,2*^, Eshini Panditharatna^1,2,3*^, Sridevi Yadavilli^1,2^, Karim Saoud^1,2,^, Sulgi Lee^1,2,15^, Augustine Eze^1,2^, M.I Almira-Suarez^4,15^, Lauren Hancock^2,5^, Erin R. Bonner^1,2,15^, Jamila Gittens^1,6^, Mojca Stampar^1^, Krutika Gaonkar^7^, Adam C. Resnick ^7^, Cassie Kline^8,16^, Cheng-Ying Ho^9^, Angela J. Waanders^10^, Maria-Magdalena Georgescu^11^, Naomi E. Rance^12^,Yong Kim^13^, Courtney Johnson^2^, Brian R. Rood^2,5^, Lindsay B. Kilburn^2,5^, Eugene I. Hwang^2,5^, Sabine Mueller^8,14^, Roger J. Packer^2^, Miriam Bornhorst^1,2#^, Javad Nazarian^1,14,15#^

**Supplementary Note 3**:

Modified SOP for external institutions

***Note: Throughout the protocol, document and photograph the following:***

- - Gross observation
  - Specimen collection/location
  - Storage condition (fixed/frozen/fresh in media)

1. Procure whole brain and photograph it.
2. Cut whole brain into half, photograph it and collect the following specimens (Fig 2)
3. Collect tumor (in the pons or other location) and process :
   1. Collect a piece of tissue (**2cm ^3^** ) and place it in Hiberanate A media tube provided
   2. Place another piece of tissue in 10% or 20% formalin for FFPE
   3. Freeze the remaining tumor sections on foil or tubes (**1cm^3^** each piece)
4. Children’s National site coordinator will arrange for shipment of the specimens
